# Difficult differentiation of a somatic symptom disorder from anterior cutaneous nerve entrapment syndrome (ACNES): a case report

**DOI:** 10.1186/s12888-019-2390-2

**Published:** 2019-12-12

**Authors:** Narifumi Yokoyama, Ryousuke Shiraki, Takashi Watanabe, Makiko Shiinoki, Michihiro Nin, Taro Shimizu, Norio Yasui-Furukori, Kazutaka Shimoda

**Affiliations:** 1 Department of Psychiatry, Dokkyo Medical University, School of Medicine, Mibu, Shimotsuga, Tochigi, 321-0293 Japan; 2 Department of Diagnostic and Generalist Medicine, Dokkyo Medical University Hospital, School of Medicine, 880 Kitakobayashi, Mibu, Shimotsuga, Tochigi, 321-0293 Japan

**Keywords:** ACNES, SSDs, Differentiating diagnosis

## Abstract

**Background:**

Somatization is regarded as psychological or emotional distress in the form of physical symptoms that are otherwise medically unexplained.

**Case presentation:**

We report a case of a patient with a somatic symptom disorder (SSD) and depression who was later diagnosed with anterior cutaneous nerve entrapment syndrome (ACNES) when Carnett’s test was positive and block anesthesia using trigger point injections dramatically improved the symptom of abdominal pain.

**Conclusion:**

We concluded that the differentiation of SSDs, such as psychogenic pain, from ACNES is very difficult. Psychiatrists should be aware of this syndrome.

## Background

Patients at all levels of health care frequently suffer from bodily complaints, such as pain in different locations of the body, fatigue, or perceived disturbances in the functions of cardiovascular, gastrointestinal, or other organ. Patients with multiple persistent physical symptoms that seem to have no apparent biological basis commonly present to primary care [1].

Generally, early (psychodynamic) models of bodily distress often imply a top-down mechanism, i.e., psychogenic activations of peripheral physiology, as the major mechanism underlying the enduring bodily symptoms. The models from the last decades predominantly imply bottom-up mechanisms where peripheral input from nociceptive and other sensors is considered to be overly amplified by central or psychosocial factors [2].

One or more somatic symptoms cause excessive thoughts, feelings, or behaviors associated with these symptoms and that are distressing or lead to a significant decrease in daily quality of life. Somatic symptom disorder (SSD) is defined as the presence of distressing somatic symptoms (criterion A) that persist for at least 6 months (criterion C) and are associated with damaging thoughts, emotions, and behavior (criterion B). Two specifiers of this condition in the Diagnostic and Statistical Manual of Mental Disorders (DSM-5) are “with predominant pain” and “persistent” [3].

In a survey, 35% experienced a medical mistake in the past 5 years involving themselves, their family, or friends; half of the mistakes were described as diagnostic errors [4]. On the other hand, inadequacies of physical examination may be a cause of medical errors [5]. The oversight caused by a failure to perform the physical examination in 63%; Special attention and skill in examining the skin and its appendages, as well as the abdomen, groin, and genitourinary area could reduce the reported oversights by half.

Here, we report a case of a patient with SSD and depression who was later diagnosed with anterior cutaneous nerve entrapment syndrome (ACNES). The patient provided written informed consent after receiving a full description of the study and consented to the publication of this report.

## Case presentation

We present a case involving a 16-year-old Japanese female who suffered from abdominal pain and a feeling of tiredness. Four months earlier, she complained of feeling tired, having abdominal pain after getting up and experiencing dyspnea. The patient was not able to go to high school and later went to a mental clinic. She went to a clinic for internal medicine, and her cortisol was 4.92 μg/mL (normal range 5.0–17.9). The adrenocorticotropic hormone load test revealed levels of 18.73 μg/mL at 30 min and 21.84 μg/mL at 60 min. Hydrocortisone 5 mg/day was administered, and she was able to wake up in the morning, but the feeling of tiredness and abdominal pain remained. Two months earlier, the patient was referred to a psychiatric outpatient clinic and was diagnosed with depression and treated with tandospirone 10 mg/day and lamotrigine 50 mg/day. She was introduced to us for psychological assessments because her abdominal pain was unchanged. We referred her to the Department of Gastrointestinal Medicine and Department of Endocrine and Metabolism in the university hospital for further physical examinations before the psychological assessments. Organic diseases in the gastrointestinal tract were excluded, and although IBS was suspected at first, this diagnosis was later disregarded. In addition, an examination of the pituitary gland magnetic resonance imaging (MRI) of the head and hormone loading tests revealed no abnormal findings. Based on the results of the physical examinations, psychogenic pain was finally suspected, and the patient was admitted to the Department of Psychiatry for detailed psychological examinations 4 months later. We switched her medication from tandospirone 30 mg/day to mirtazapine 30 mg/day. Her depressive symptoms improved, but her abdominal pain remained. Because Carnett’s test was positive but no abnormality was found from a simple abdominal computed tomography (CT) scan, we suspected ACNES in addition to depression and referred her to Department of Diagnostic and Generalist Medicine. Block anesthesia using trigger point injections dramatically improved the abdominal pain. We concluded that her diagnosis was ACNES, and the patient was discharged. She underwent dissection of the left intercostal nerve and anterior sheath in the abdomen 6 months later. The patient has not complained of any abnormal pain, and her mental condition has been stable since the dissection.

## Discussion and conclusion

ACNES causes chronic abdominal pain [6]. However, both psychiatrists and physicians are still unaware of this diagnosis. The literature on the prevalence of ACNES in the pediatric population is very scarce. ACNES has only recently been described in children [7–10]. Furthermore, little information on ACNES exists in the psychiatric field. Pain without an organic reason is frequently diagnosed as SSD, as in our case.

ACNES leads to abdominal wall pain, which is believed to be because the superficial branches of the intercostal thoracic nerves are entrapped between the abdominal muscles and results in pain in the specific location of entrapment. Therefore, ACNES can mimic an intra-abdominal source of pain, such as appendicitis. Antidepressants are not effective for managing ACNES because the origin of the pain is mechanical. Instead, trigger point injections and ultrasound-guided nerve blocks are effective in treating ACNES. Applegate et al. described injecting a local anesthetic with or without a corticosteroid at the point of maximal tenderness into the aponeurosis of the rectus abdominis [7]. Using ultrasound guidance increases the accuracy of the nerve block and decreases the risk of entering the peritoneal cavity.

There have been only two studies that reported cases of ACNES previously assumed to be of psychiatric origin. The diagnosis of ACNES took 2 years in a case described by Thome and Egeler [11]. In the other case, 8 months was needed to finalize a diagnosis of ACNES [12]. Owing to its rarity and difficulty to diagnose, ACNES often presents as a chronic pain problem, with 30 to 35% of patients reporting pain starting more than 1 year before the initial presentation [13]. To reproduce the pain associated with anterior cutaneous nerve entrapment, Carnett’s sign should be evaluated [13, 14]. The role of this sign is to differentiate somatic pain from visceral abdominal pain. To perform the Carnett’s test, the patient is asked to lie down and point to the specific area of abdominal pain. The examiner presses on the point of maximal tenderness with one finger as the patient raises both legs or raises his or her head. If the pain worsens, then Carnett’s sign is positive.

Therefore, we concluded that the differentiation of SSDs, such as psychogenic pain, from ACNES is very difficult. Not only psychiatrists but also physicians should be aware of this syndrome.

## Data Availability

All data generated or analyzed during this study are included in this published article.

## Abbreviations

ACNES: Anterior cutaneous nerve entrapment syndrome
CT: Computed tomography
DSM-5: Diagnostic and statistical manual of mental disorders
MRI: Magnetic resonance imaging
SSD: Somatic symptom disorder
